# Taxonomic and nomenclatural notes on the genera *Themus* Motschulsky and *Lycocerus* Gorham (Coleoptera, Cantharidae)

**DOI:** 10.3897/zookeys.340.5470

**Published:** 2013-10-04

**Authors:** Yuxia Yang, Andreas Kopetz, Xingke Yang

**Affiliations:** 1 College of Life Sciences, Hebei University, Baoding 071002, Hebei Province, China; 2 Im Semmichbache 14, D-99334 Eischleben, Germany; 3 Key Laboratory of Zoological Systematics and Evolution, Institute of Zoology, Chinese Academy of Sciences, Beijing 100101, China

**Keywords:** Coleoptera, Cantharidae, *Themus*, *Lycocerus*, synonym, homonym, new name, restoration name, new combination, new status, resurrection

## Abstract

The following taxonomic or nomenclatural changes are proposed: *Themus* (s.str.) *regalis* (Gorham, 1889), **nom. rest.**; *Themus* (s.str.) *scutulatus* Wittmer, 1983 = *Themus* (s.str.) *hmong* Kazantsev, 2007, **syn. n.**; *Themus (Telephorops) coelestis* (Gorham, 1889) = *Themus violetipennis* Wang & Yang, 1992, **syn. n.**; *Themus (Telephorops) uniformis* Wittmer, 1983, **stat. n.** = *Themus (Telephorops) cribripennis* Wittmer, 1983, **syn. n.**; *Themus (Haplothemus) licenti* Pic, 1938, **stat. rev.**, resurrected from synonymy with *Themus coriaceipennis* (Fairmaire, 1889); *Lycocerus aenescens* (Fairmaire, 1889) = *Lycocerus tcheonanus* (Pic, 1922), **syn. n.**; *Lycocerus asperipennis* (Fairmaire, 1891) = *Lycocerus wangi* (Švihla, 2004), **syn. n.**; *Lycocerus borneoensis* **nom. n.** for *Athemellus atricolor* (Wittmer, 1972); *Lycocerus bilineatus* (Wittmer, 1995) = *Lycocerus amplus* (Wittmer, 1995), **syn. n.**; *Lycocerus fairmairei* **nom. n.** et **stat. rev.** for *Athemus dimidiaticrus* (Fairmaire, 1889), originally in *Telephorus*, resurrected from synonymy with *Lycocerus orientalis* (Gorham, 1889); *Lycocerus confossicollis* (Fairmaire, 1891), **comb. n.** hereby transferred from *Cantharis* = *Lycocerus multiimpressus* (Wittmer, 1997), **syn. n.**; *Lycocerus inopaciceps* (Pic, 1926) = *Athemus (Athemellus) bimaculicollis* (Švihla, 2005), **syn. n.**; *Lycocerus nigratus* **nom. n.** for *Lycocerus nigricolor* (Wittmer, 1972), originally in *Podabrinus*; *Lycocerus plebejus* (Kiesenwetter, 1874) = *Lycocerus brunneonotaticeps* (Pic, 1922), **syn. n.** = *Cantharis rufonotaticeps* Pic, 1921 **syn. n.**; *Lycocerus swampingatus* (Pic, 1916), **comb. n.**, hereby transferred from *Cantharis*. The neotypes of *Themus violetipennis* Wang & Yang, 1992 and *Athemus* (s.str.) *maculithorax* Wang & Yang, 1992 are designated respectively.

## Introduction

This study presents some further taxonomic and nomenclatural clarification in the cantharid genera *Themus* Motschulsky, 1857 and *Lycocerus* Gorham, 1889, based on the examination of type specimens. See Wittmer (1961, 1969, 1972, 1983a, 1995) for prior taxonomic changes. The present work primarily focuses on the Chinese species.

## Material and methods

The aedeagi and the abdominal sternite VIII of female were dissected under a stereoscopic microscope, cleared in 10% KOH solution for several minutes, then placed in a droplet of glycerol and examined under a compound light microscope. Photographs of the type specimens were taken with a Canon 450D camera equipped with an EF 100mm f/2.8 USM lens. Line drawings were made with the aid of camera lucida attached to a Leica MZ12.5 stereomicroscope, and edited in the CorelDRAW 12 and Adobe Photoshop 8.0.1.

In the literature citations, the square brackets “[ ]” are used for my remarks and addenda. The type specimens are quoted verbatim, “[p]” indicated that the following data are printed and “[h]” that they are handwritten, and the quotation marks are used to separate data from different labels and a backslash “\” to separate data from different lines of the same label. The additional specimens are transliterated from Chinese labels, except those originally in English and cited in quotation marks.

The following collection codens are used in the text:

HBUM Hebei University Museum, Baoding, China;

IZAS Institute of Zoology, Chinese Academy of Sciences, Beijing, China;

MNHN Muséum national d’Histoire naturelle, Paris, France;

NHMB Naturhistorisches Museum Basel, Switzerland;

NMPC Narodni muzeum, Praha, Czech Republic.

## Taxonomic account

### Themus (s.str.) regalis

(Gorham, 1889) nom. rest.

Telephorus regalis Gorham 1889: 103. [Synonymized with *Themus imperialis* (Gorham, 1889) by Wittmer 1983a: 215.]
Telephorus imperialis Gorham 1889: 102, t. 10, fig. 8. [Primary homonym, preoccupied by *Telephorus imperialis* Redtenbacher, 1867.]
Cantharis imperator Pic 1906: 81. [Replacement name for *Telephorus imperialis* Gorham, 1889, nec Redtenbacher 1867.]
Themus imperator : Jacobson 1911: 675.
Themus regalis : Jacobson 1911: 675.
Themus (s.str.) *imperialis*: Wittmer 1983a: 215.

#### Distribution.

China, Vietnam.

#### Remarks.

*Telephorus imperialis* Gorham, 1889 is a primary homonym and preoccupied by *Telephorus imperialis* Redtenbacher, 1867, so the former is permanently invalid (ICZN, 4^th^ ed., article 57.2) and should be replaced by the next oldest available name among its synonyms (ICZN, 4^th^ ed., article 23.3.5), that is, *Themus* (s.str.) *regalis* (Gorham, 1889) should be restated as the valid name for this species.

### Themus (s.str.) scutulatus

Wittmer, 1983

Themus rufoscutus Pic 1926a: 35. [Secondary homonym, preoccupied by *Themus rufoscutus* (Pic, 1922), originally described in *Cantharis*.]
Themus (s.str.) *scutulatus*Wittmer 1983a: 208. [Replacement name for *Themus rufoscutus* Pic, 1926, nec Pic 1922.]
Themus (s.str.) *hmong*Kazantsev 2007: 54. [Replacement name for *Themus rufoscutus* Pic, 1926, nec Pic 1921 [1922].] syn. n.

#### Distribution.

Vietnam.

#### Remarks.

This species was originally described as *Themus rufoscutus* Pic, 1926 (located in Vietnam), which became a junior secondary homonym of *Themus rufoscutus* (Pic, 1922) (located in Yunnan, China) since the taxonomic status of the latter was changed by Wittmer (1983a), so the former was replaced by *Themus* (s.str.) *scutulatus* in the latter study. However, this nomenclature change was neglected by Kazantsev (2007), so that *Themus rufoscutus* Pic, 1926 was replaced again by *Themus* (s.str.) *hmong*. In the same work (Kazantsev and Brancucci 2007), the distribution of this species was recorded occurring in both Vietnam and China (Yunnan). Obviously, *Themus* (s.str.) *hmong* is a junior objective synonym of *Themus* (s.str.) *scutulatus* (ICZN, 4^th^ ed., article 72.7), which should be restricted to Vietnam and excluded from the Chinese fauna at the moment.

### Themus (Telephorops) coelestis

(Gorham, 1889)

http://species-id.net/wiki/Themus_coelestis

Telephorus coelestis Gorham 1889: 104, t. 10, fig. 7.
Themus coelestis : Jacobson 1911: 675.
Themus rugosus Pic 1929: 8. [Synonymized by Wittmer 1983a: 197.]
Themus (Telephorops) coelestis : Wittmer 1983a: 197, figs. 1, 59.
Themus violetipennis Wang and Yang 1992: 265, fig 2.
Themus (s.str.) *violetipennis*: Švihla 2008: 186. syn. n.

#### Type material examined.

*Telephorus coelestis*: Lectotype ♂ (NHMB): without locality information, [h]“coelestis ♂”, [h]“♂”, [h]“Themus \ (Telephorops) \ coelestis \ (Gorh.) \ det. W. Wittmer”, [h]“Type”, [p]“LECTOTYPUS”, [p]“Naturhist. \ Museum Basel \ coll. W. Wittmer”, [p]“CANTHARIDAE \ CANTH00001277”. Paralectotype: 1♀ (MNHN): [p]“Kiukiang \ June, 1887 \ A. E. Pratt”, [h]“coelestis \ Gorh.”, [h]“Themus \ (Telephorops) \ coelestis \ (Gorh.) \ det. W. Wittmer”, [h]“TYPE”, [p]“PARALECTOTYPUS”.

*Themus rugosus*: Holotype ♀ (MNHN): [h]“Fokien”, [h]“Themus \ rugosus \ n. sp.”, [h]“Themus \ (Telephorops) \ coelestis \ (Gorh.) \ det. W. Wittmer”, [h]“type”, [p]“TYPE”.

#### Neotype designation.

*Themus violetipennis*: Neotype ♀ (here designated, IZAS): “湖南永顺杉木河林场 \ 600m \ 中国科学院” [Hunan, Yongshun, Shanmuhe forestry station \ 600m], “1988.VIII.4 \ 采集者：王书永” [4.VIII.1988 \ leg. Shu-Yong Wang].

#### Additional material examined.

CHINA: Shaanxi: 2♀♀ (IZAS): Foping, 16.VIII.2007, leg. Yu-Xia Yang. Gansu: 1♂ (IZAS): Kangxian, Baiyunshan, 1250–1750m, 12.VII.1998, leg. Shu-Yong Wang; 1♀ (IZAS): Kangxian, Heimaguan, 1450–1550m, 13.VII.1998, leg. De-Cheng Yuan. Henan: 1♂ (IZAS): Jigongshan, 700m, 14.VII.2001, leg. Si-Qin Ge; 1♀ (IZAS): Tongbaishan, 500m, 16.VII.2001, leg. Si-Qin Ge; 1♀ (IZAS): Neixiang, Baoyunman, 21.VII.2001, leg. Fu-Qiang Chen. Anhui: 2♂♂ (NHMB): “Dabieshan, 65km SW Huoshan, 1400m, 21.–24.VI.1995, leg. Bolm”. Zhejiang: 1♀ (IZAS): Xitianmushan, 23.VI.1998, leg. Ming-Shui Zhao. Hubei: 1♂, 1♀ (NHMB): “Hupeh, Lichuan Distr., Suisapa, 1000m, 22.VII.1948, Gressitt&Djou Collra”; 1♂ (NHMB): same data, 24.VII.1948; 1♂ (NHMB): same data, 29.VII.1948; 2♂♂, 1♀ (NHMB): same data, 31.VII.1948; 1♂, 1♀ (NHMB): same data, 4.VIII.1948; 1♂ (IZAS): Hefeng, Fenshuiling, 1400m, 1.VIII.1981, leg. Long-Long Yang; 1♀ (IZAS): Hefeng, Shayuan, 30.VII.1989, leg. Shu-Yong Wang. Jiangxi: 1♂ (IZAS): Jiulianshan, Huangniushi, 19.VI.1975, leg. You-Wei Zhang; 1♀ (IZAS): Longnan, Jiulianshan, 17.VI.1975, leg. You-Wei Zhang. Hunan: 1♂, 1♀ (NHMB): “Yon-ping, 12.VI.1917”; 1♂ (NHMB): same data, “Yon-ping, 14.VI.1917”; 1♂ (IZAS): Sangzhi, Tianpingshan, 700–1450m, 14.VIII.1988, leg. Shu-Yong Wang. Fujian: 1♂ (IZAS): Chong’an, Xingcun, Xianfengling, 1170m, 14.VII.1960, leg. Cheng-Lin Ma; 1♀ (IZAS): Dehua, Chengguan, 510–550m, 1.VI.1960, leg. Fu-Ji Pu; 11 spec. (NHMB): “Fukien, Kuatun, 15.VIII.1946, Tschung-Sen leg.”; 5 spec. (NHMB): “Kuatun, 26.VII.1946”; 6 spec. (NHMB): same data, 11.VII.1946; 5 spec. (NHMB): same data, 16.VIII.1946; 4 spec. (NHMB): same data, 18.IX.1946. Hainan: 1♀ (IZAS): Wanning, 10m, 9.VI.1960, leg. Chang-Qing Li. Guangxi: 1♂ (IZAS): Longsheng, Tianpingshan, 740m, 17.VI.1963, leg. Shu-Yong Wang; 1♀ (IZAS): Maoershan, Tongmujiang, 800m, 15.VII.1985, leg. Su-Bai Liao. Sichuan: 1♂, 1♀ (IZAS): Youyang, 780m, 15.VII.1989, leg. Shu-Yong Wang. Guizhou: 1♂ (NHMB): “Kouy-Tchéou”; 1♂ (IZAS): Fanjingshan, Huguosi, 1350m, 3.VIII.2001, leg. Qiong-Zhang Song; 1♀ (IZAS): Fanjingshan, Huixiangping, 1600m, 2.VIII.2001, leg. Kang-Zhen Dong.

#### Distribution.

China (Shaanxi, Gansu, Henan, Anhui, Zhejiang, Hubei, Jiangxi, Hunan, Fujian, Hainan, Guangxi, Sichuan, Guizhou).

#### Remarks.

According to the original publication, the types of *Themus violetipennis* Wang & Yang, 1992 were deposited in the IZAS and China Agriculture University, Beijing, China (CAUB), but our search of the types in the two Chinese museums have been long, repeated and with no results. The original description of *Themus violetipennis* was in accord with the standard of that time but insufficient considering the present level, and neotype allows us to satisfy a better comparision. Fortunately, a female specimen, which was collected at the same locality and date as that of one paratype designated by Wang and Yang (1992), was found in IZAS during our study. Its morphological characters are consistent with the original description, so it is designated as the neotype here, in order to clarify the taxonomic status of this species (ICZN, 4^th^, article 75.3). Furthermore, a careful examination of the types shows that *Themus violetipennis* Wang & Yang, 1992 is a junior synonym of *Themus (Telephorops) coelestis* (Gorham, 1889), which is widely distributed in China based on the data from a large series of examined specimens.

### Themus (Telephorops) uniformis

Wittmer, 1983 stat. n.

Figs 1–2

Themus (s.str.) *bitinctus uniformis*Wittmer 1983a: 218, fig. 30.
Themus (s.str.) *cribripennis*Wittmer 1983b: 151, figs. 46, 49.
Themus (Telephorops) bitinctus uniformis : Švihla 2008: 187.
Themus (Telephorops) cribripennis : Švihla 2008: 187. syn. n.

#### Type material examined.

*Themus* (s.str.) *bitinctus uniformis*: Holotype ♂ (NHMB): [p]“Yen-ping, China \ VII.21. 1917 \ Ac. 5148”, [h]“bitinctus \ uniformis”, [p]“HOLOTYPUS”, [p]“Naturhist. \ Museum Basel \ coll. W. Wittmer”, [p]“CANTHARIDAE \ CANTH00000449”.

*Themus* (s.str.) *cribripennis*: Holotype ♂ (MNHN): [p]“Taihorinsho \ Formosa \ Sauter, VIII_ 7_09”, [h]“Cantharis \ davidis Fairm.”, [h]“Themus (s.str.) \ cribripennis \ Wittm. \ det. W. Wittmer”, [p]“HOLOTYPUS”, [h]“136”. Paratype: 1♀ (NHMB): [p]“Suisharyo \ Formosa \ H. Sauter, X.1911”, [h]“Themus s.str. \ cribripennis \ Wittm. \ det. W. Wittmer”, [p]“PARATYPUS”, [p]“CANTHARIDAE \ CANTH00002654”.

**Figures 1–9.**
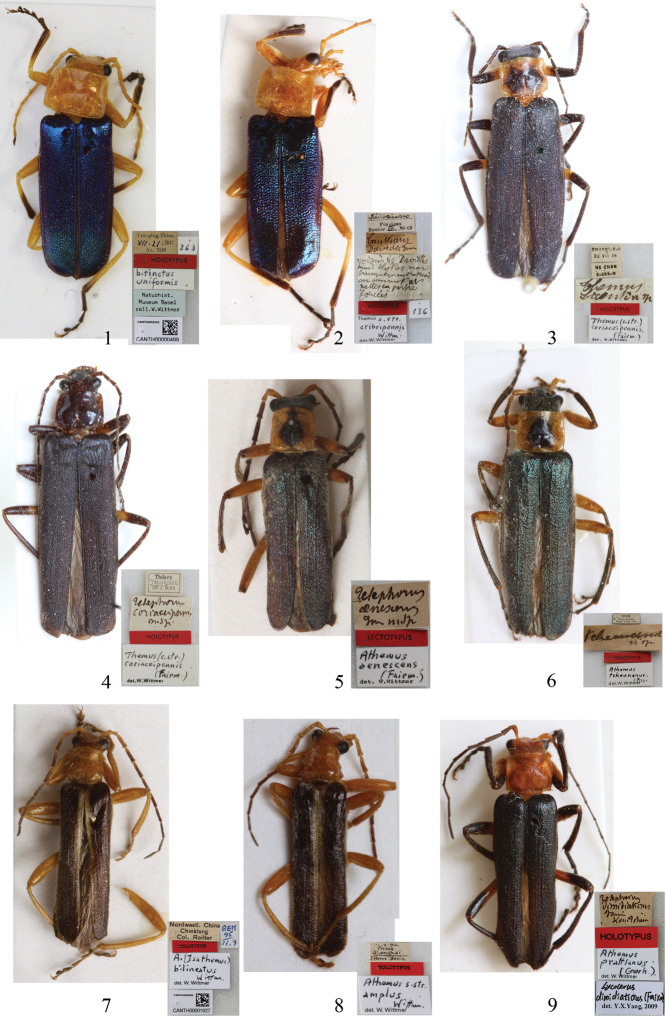
Habitus, dorsal view **1** Holotype of *Themus* (s.str.) *bitinctus uniformis* Wittmer, 1983 **2** Holotype of *Themus* (s.str.) *cribripennis* Wittmer, 1983 **3** Holotype of *Themus licenti* Pic, 1938 **4** Holotype of *Telephorus coriaceipennis* Fairmaire, 1889 **5** Lectotype of *Podabrus aenescens* Fairmaire, 1889 **6** Holotype of *Cantharis tcheonana* Pic, 1922 **7** Holotype of *Athemus (Isathemus) bilineatus* Wittmer, 1995**8** Holotype of *Athemus* (s.str.) *amplus* Wittmer, 1995 **9** Holotype of *Telephorus dimidiaticrus* Fairmaire, 1889. **1–2, 5, 7–8, 9** male **3–4, 6** female.

#### Distribution.

China (Fujian, Taiwan).

#### Remarks.

Having examined the holotypes of *Themus cribripennis* Wittmer, 1983b and *Themus bitinctus uniformis* Wittmer, 1983a, we were unable to find differences justifying their separation, which has led us to consider all the examined specimens of both nominal species to be conspecific. Therefore, we synonymized *Themus cribripennis* under *Themus bitinctus uniformis*. Furthermore, *Themus bitinctus uniformis* should be upgraded to the specific rank, because it is obviously different from *Themus bitinctus* Wittmer, 1982 (located in Vietnam) in the aedeagus, except the difference in the elytra coloration from the latter.

### Themus (Haplothemus) licenti

Pic, 1938 stat. rev.

Fig. 3

Themus licenti Pic 1938a: 161. [Synonymized with *Themus coriaceipennis* (Fairmaire, 1889) by Wittmer 1983a: 224.]
Themus coriaceipennis (Fairmaire, 1889): Wittmer 1983a: 224, figs 43, 111. [Misidentification.]

#### Type material examined.

*Themus licenti*: Holotype ♀ (MNHN): [p]“CHANSI. S.O. \ 22.VII.35”, [p]“HO CHAN \ 2,255m”, [h]“Themus \ licenti n. sp.”, [h]“Themus (s.str.) \ coriaceipennis \ (Fairm.) \ det. W. Wittmer”, [p]“HOLOTYPUS”.

*Telephorus coriaceipennis*: Holotype ♀ (MNHN): [p]“Thibet \ Tàtsiénloù \ M. F. Biet”, [h]“Telephorus \ coriaceipennis \ n. sp.”, [h]“Themus (s.str.) \ coriaceipennis \ (Fairm.) \ det. W. Wittmer”, [p]“HOLOTYPUS”.

#### Additional material examined.

CHINA: Henan: 1♂, 1♀ (IZAS): Lushi, Jihe forestry station, 1200m, 20.VII.2001, leg. Kang-Zhen Dong. Sichuan: 5♂♂, 1♀ (NHMB): “Szechuen, Yao Gi, nr Mupin, 7400ft., 15.VII.1929, D. C. Graham”; 1♂, 1♀ (NHMB): “Mu San Tsai, 10km NW Weichow, 8700ft., 26.–28.VI.1933, D. C. Graham”; 1♂, 1♀ (IZAS): Luding, Xinxing, Yanzigou, 1560m, 7.VIII.2004, leg. Ming Bai.

#### Distribution.

China (Henan, Shaanxi, Sichuan).

#### Remarks.

*Themus licenti* Pic, 1938a was synonymized with *Themus coriaceipennis* (Fairmaire, 1889) by Wittmer (1983a). However, examination of the holotypes of both nominal species shows that they are different species. *Themus licenti* is obviously different from *Themus coriaceipennis* in the following characters: head (Fig. 3) width across eyes wider than anterior margin of pronotum, pronotum reddish brown with a large black marking in middle, abdominal sternite VIII of female (see Wittmer 1983a: fig. 111) deeply concaved on both sides of the middle emargination of posterior margin; while in *Themus coriaceipennis* (Fig. 4), head width across eyes narrower than anterior margin of pronotum, pronotum uniformly dark brown, without any black marking, abdominal sternite VIII of female (Fig. 22) slightly concaved on both sides of the middle emargination of posterior margin. Therefore, we suggest *Themus licenti* Pic, 1938a be resurrected from synonymy with *Themus coriaceipennis* (Fairmaire, 1889).

### Lycocerus aenescens

(Fairmaire, 1889)

http://species-id.net/wiki/Lycocerus_aenescens

Figs 5−6

Podabrus aenescens Fairmaire 1889: 40.
Cantharis tcheonana Pic 1922: 31.
Themus angusticollis Pic 1955: 16. [Secondary homonym, preoccupied by *Athemus angusticollis* (Gorham, 1882), originally described in *Telephorus*. Synonymized with *Athemus tcheonanus* (Pic, 1922) by Wittmer 1995: 211.]
Athemus angustithorax Wittmer 1975: 260. [Replacement name for *Themus angusticollis* Pic, 1955, nec Gorham 1882.]
Athemus tcheonanus : Wittmer 1975: 260; 1995: 211, figs. 42, 43, 168, 169.
Athemus aenescens : Wittmer 1982: 341.
Lycocerus aenescens : Kazantsev and Brancucci 2007: 249.
Lycocerus tcheonanus : Kazantsev and Brancucci 2007: 254. syn. n.

#### Type material examined.

*Podabrus aenescens*: Lectotype ♂ (MNHN): without locality information, [h]“Telephorus aenescens \ Frm., n. sp.”, [h]“Athemus \ aenescens \ (Fairm.) \ det. W. Wittmer”, [p]“LECTOTYPUS”. Paralectotype: 1♀ (MNHN): [p]“Kouy-Tchèu”, [p]“PARALECTOTYPUS”.

*Cantharis tcheonana*: Holotype ♀ (MNHN): [p]“CHINE \ Kouy-Tchèou \ Koúy Yang Foú”, [h]“tcheonana \ n. sp.”, [h]“Athemus \ tcheonanus \ (Pic) \ det. W. Wittmer”, [p]“HOLOTYPUS”.

*Themus angusticollis*: Holotype ♂ (MNHN): [p]“Chasseurs Indigenes \ des Missionnaires \ de Ta-tslen-Lou \ 1906”, [h]“Themus \ angusticollis \ n. sp.”, [h]“Athemus s.str. \ angustithorax \ Wittm. \ det. W. Wittmer”, [h]“Athemus s.str. \ tcheonanus \ (Pic) \ det. W. Wittmer”, [p]“HOLOTYPUS”.

#### Additional material examined.

CHINA: Guangxi: 1♂, 1♀ (NHMB): “Koung-Si-Hien, alt. 2100m”. Sichuan: 1♂ (NHMB): “Moxi, Gongashan mts., 1650m, 28.VI–2.VII.1994, leg. Bolm”. Guizhou: 1♂ (MNHN): “Kouy-Tchèu, Koúy Yang Foú”; 2♀♀ (MNHN): “Kouy-Tchèu”; 1♀ (MNHN): “Kouei-Tcheou”; 5♀♀ (MNHN): “Kuoy-Tcheou, Gan chouem, Min y fou et Tchen-Fong, Tchéou, 1918, P. Cavalerie”; 1♀ (MNHN): “Kouy-Tchèu, 1921, Cavaleri”; 1♀ (MNHN): “Kouy-Tchèu, Reg. De Pin-fa, 1908, Père Cavaleri”. Yunnan: 1♂ (MNHN): “Tse Kou, 1895, R. P. Dubernard”; 1♂, 1♀ (IZAS): Dali, Cangshan, 30.V.1955, leg. Xing-Chi Yang; 8 spec. (NHMB): “Cangshan mts., E slope, 2000–2500m, 25°42'N, 100°08'E, 21.VI.1992, leg. Vit Kubáň”; 12 spec. (NHMB): “Dali, 19.–21.V.1993, leg. R. Cervenka”; 2♂♂, 1♀ (NHMB): “Dali, 1600–2000m, 5.–8.VII.1990, leg. L. M. Bocák”; 1♂, 2♀♀ (NHMB): “Dali, 1.–7.VI.1994, B. Šiška et T. Spevár”.

#### Distribution.

China (Guangxi, Sichaun, Guizhou, Yunnan).

#### Remarks.

Having examined the holotypes of *Lycocerus aenescens* (Fairmaire, 1889) and *Lycocerus tcheonanus* (Pic, 1922), we could not find any difference to justify their separation, so we synonymize *Lycocerus tcheonanus* under *Lycocerus aenescens*.

### Lycocerus asperipennis

(Fairmaire, 1891)

http://species-id.net/wiki/Lycocerus_asperipennis

Telephorus asperipennis Fairmaire 1891: ccviii.
Cantharis limbatipennis Pic 1906: 83. [Synonymized by Wittmer 1995: 256.]
Cantharis asperipennis : Jacobson 1911: 679.
Cantharis stötzneri Pic 1926b: 154. [Synonymized by Wittmer 1995: 256.]
Athemus stötzneri : Pic 1933: 4.
Athemus limbatipennis : Wittmer 1972a: 106.
Athemus (s.str.) *maculithorax*Wang and Yang 1992: 264, fig. 1. [Secondary homonym, preoccupied by *Athemus maculithorax* (Wittmer, 1972), originally described in *Athemellus*.]
Athemus (s.str.) *asperipennis*: Wittmer 1995: 256, figs. 113, 114.
Athemus (s.str.) *wangi*Švihla 2004: 183 [Replacement name for *Athemus maculithorax* Wang & Yang, 1992, nec Wittmer 1972.]
Lycocerus asperipennis : Kazantsev and Brancucci 2007: 249.
Lycocerus wangi : Kazantsev and Brancucci 2007: 254. syn. n.

#### Type material examined.

*Telephorus asperipennis*: Lectotype ♀ (MNHN): [h]“Chang-yang”, [h]“Telephorus \ asperipennis \ Fairm. \ Changyang”, [h]“Athemus \ asperipennis \ (Fairm.) \ det. W. Wittmer”, [p]“LECTOTYPE”. Paralectotype: 1♀ (MNHN): [h]“Chang-yang”, [p]“PARALECTOTYPE”.

*Cantharis limbatipennis*: Lectotype ♀ (MNHN): [h]“Yunnan \ (China)”, [h]“C. limbatipennis \ Pic”, [h]“limbatipennis \ Pic”, [h]“von \ asperipennis \ Frm.”, [h]“Athemus \ asperipennis \ (Frm.) \ det. W. Wittmer”, [h]“type”, [p]“TYPE”, [p]“LECTOTYPE”. Paralectotype: 1♀ (MNHN): [h]“Yunnan”, [p]“PARALECTOTYPE”.

*Cantharis stötzneri*: Lectotype ♂ (MNHN): [p]“Szetschwan \ Kwanhsien \ Exp. Stötzner”, [h]“stötzneri \ n. sp.”, [h]“Athemus \ asperipennis \ (Frm.) \ det. W. Wittmer”, [p]“LECTOTYPE”. Paralectotypes: 1♂, 5♀♀ (MNHN): same data to lectotype, [p]“PARALECTOTYPE”.

#### Neotype designation.

*Athemus maculithorax*: Neotype ♂ (here designated, IZAS): [p]“湖北兴山龙门河 \ 1300m” [Hubei, Xingshan, Longmenhe \ 1300m], [p]“1993.VI.21 \ 采集者：黄润质” [21.VI.1993 \ leg. Run-Zhi Huang].

#### Additional material examined.

CHINA: Gansu: 1♂ (IZAS): Wenxian, Qiujiaba, 2360–2650m, 29.VI.1998, leg. De-Cheng Yuan; 1♀ (IZAS): same data, 30.VI.1998. Shanxi: 1♀ (IZAS): “Shansi, Kwashan, 9.VI.1936”. Shaanxi: 2♂♂ (NHMB): “Danfeng-NE env., 900–1500m, 33°45–52'N, 110°22–37'E, 28.–29.V.1995, leg. L. R. Businský”; 3♂♂, 1♀ (NHMB): “Qinling Mts.-N. slpoe, Huxian Co., 1300–1600m, 33°50'N, 108°26'E, 12.–13.VI.1995, leg. L. R. Businský”. Henan: 3♀♀ (IZAS): Lushi, Jihe forestry station, 20.VII.2001, leg. Kang-Zhen Dong. Hubei: 1♂ (NHMB): “Shennongjia, Yanzi Pass, 2200m, 31°43'N, 110°28'E, 23.–26.VI.1995, leg. L. R. Businský”; 1♀ (NHMB): “Dashennongjia massif-E slpoe, 31°24–30'N, 110°21–24'E, 2000m, 28.VI–7.VII.1995, leg. L. R. Businský”; 1♂, 1♀ (IZAS): Foping, Liangfengya, 1750–2150m, 28.VI.1999, leg. YAO Jian. Sichuan: 10 spec. (NHMB): “Mt. Emei, 500–1200m, 29°30'N, 103°20'E, 4.–18.V.1989, leg. S. J. Kolibáč”; 12 spec. (NHMB): “Mt. Emei, 600–1050m, 5.–19.V.1989, leg. Lad. Bocák”; 25 spec. (NHMB): “Kwanhsien, Exp. Stötzner”; 1♂, 1♀ (NHMB): “Chengtu, 1700m, 1.–2.V.1933, D.C. Graham”; 1♂, 1♀ (NHMB): “Kuausien, 1934, D.C. Graham”; 1♂, 1♀ (NHMB): “Yaogi nr. Mupin, 8000ft, 14.–18.VI.1929, D.C. Graham”; 1♀ (NHMB): “Shikaizi, Mt. Omei, 4500ft, 1945, D.C. Graham”; 1♂ (IZAS): Mt. Emei, Baoguosi, 550–750m, 27.IV.1957, leg. Fu-Xing Zhu; 1♀ (IZAS): Mt. Emei, Qingyinge, 800–1000m, 20.IV.1957, leg. Fu-Xing Zhu; 1♀ (IZAS): same locality, 30.IV.1957, leg. Ke-Ren Huang; 1♂ (IZAS): same locality, 24.IV.1957, leg. Zong-Yuan Wang.

#### Distribution.

China (Gansu, Shanxi, Shaanxi, Henan, Hubei, Sichuan, Yunnan).

#### Remarks.

The neotype is designated for *Athemus* (s.str.) *maculithorax* Wang & Yang, 1992 here, according to the loss of the type, and for allowing a comparision based on most of the criteria (ICZN, 4^th^, article 75.3). The latter’s name was replaced by *Athemus wangi* by Švihla (2004) and now is placed in *Lycocerus* (Kazantsev and Brancucci 2007). Having examined the lectotype of *Lycocerus asperipennis* and a large series of additional specimens from China, we suggest *Lycocerus wangi* is a junior synonym of *Lycocerus asperipennis*, since that we could not find any difference to justify their separation.

### Lycocerus borneoensis

Y. Yang & X. Yang nom. n.

Podabrinus atricolor Pic 1938a: 158.
Pseudoabsidia atricolor : Wittmer 1969: 128.
Athemellus atricolor : Wittmer 1972a: 126 [inc. sed.]; Delkeskamp 1977: 47. [Secondary homonym, preoccupied by *Lycocerus atricolor* (Pic, 1922), originally described in *Cantharis*.]

#### Distribution.

Borneo.

#### Etymology.

The new name is derived from this species’ type locality “Borneo”.

#### Remarks.

This species was located in Borneo and originally described in *Athemellus* Wittmer, 1972, which was synonymized with *Lycocerus* Gorham, 1889 by Okushima (2005), so it should be placed in the latter genus for the time being. Because of this change, this species and *Lycocerus atricolor* (Pic, 1922) (originally in *Athemus*) become secondary homonyms and the junior is invalid (ICZN, 4^th^, article 57.3.1), so its name is replaced by *Lycocerus borneoensis* nom. n. here.

### Lycocerus bilineatus

(Wittmer, 1995)

http://species-id.net/wiki/Lycocerus_bilineatus

Figs 7–8

Athemus (Isathemus) bilineatus Wittmer 1995: 275, figs. 140, 141.
Athemus (s.str.) *amplus*Wittmer 1995: 278, figs. 146, 147, 203.
Lycocerus amplus : Kazantsev and Brancucci 2007: 249. syn. n.
Lycocerus bilineatus : Kazantsev and Brancucci 2007: 249.

#### Type material examined.

*Athemus (Isathemus) bilineatus*: Holotype ♂ (NHMB): [p]“Nordwestal. China \ Chinkiang \ Col. Reitter”, [h]“REM \ 95 \ 11.9”, [h]“A. (Isathemus) \ bilineatus \ Wittm., \ det. W. Wittmer”, [p]“HOLOTYPUS”, [p]“CANTHARIDAE \ CANTH00001927”.

*Athemus* (s.str.) *amplus*: Holotype ♂ (MNHN): [h]“V.1917 \ China \ Shanghai \ J. Hervé-Bdzin”, [h]“Athemus s.str. \ amplus \ Wittm. \ det. W. Wittmer”, [p]“HOLOTYPUS”. Paratypes: 3♂♂, 2♀♀ (MNHN): [h]“Shanghai”, [p]“PARATYPUS”; 1♂ (NHMB): [p]“Shanghai \ V.1917 \ J. Hervé-Bdzin”, [p]“PARATYPUS”, “CANTHARIDAE \ CANTH00001340”; 1♀ (NHMB): [p]“Shanghai \ V \ 1917, J. Hervé-Bdzin”, [p]“PARATYPUS”, [p]“CANTHARIDAE \ CANTH00002072”; 1♂ (NHMB): [p]“Zi-ka-wei \ 20.IV.1924”, [p]“PARATYPUS”, [p]“CANTHARIDAE \ CANTH00001986”.

#### Additional material examined.

CHINA: Shanghai: 1♂, 2♀♀ (IZAS): “Shanghai, 19.VI.1947, Marist Brothers”; 1♂ (IZAS): “Zi-ka-wei”, 20.IV.1924. Jiangxi: 2♂♂, 1♀ (IZAS): Jiangxi.Hubei: 2♂♂ (IZAS): Xingshan, Xiakou, 140m, 2.V.1994, leg. Xing-Ke Yang; 2♀♀ (IZAS): Zigui, Jiulingtou, 110m, 1.V.1994, leg. Wen-Zhu Li.

#### Distribution.

China (Jiangsu, Shanghai, Jiangxi, Hubei).

#### Remarks.

Both *Lycocerus amplus* (Wittmer, 1995) and *Lycocerus bilineatus* (Wittmer, 1995) were originally described in *Athemus* and assigned to different subgenera. In the original manuscript (Wittmer 1995), *Lycocerus bilineatus* was described on a single male type, so it made no sense that it was attributed to the subgenus *Isathemus* because of no female available. Furthermore, having examined the holotypes of both nominal species and some paratypes, as well as a series of additional specimens, we could not find any difference justifying their separation, even in the tarsal claws, which is the character to distinguish the subgenera *Athemus* and *Isathemus* (Wittmer 1995). Consequently, we synonymized *Lycocerus amplus* under *Lycocerus bilineatus*.

### Lycocerus fairmairei

Y. Yang & X. Yang nom. n. et stat. rev.

Figs 9 , 19−21 , 23

Telephorus dimidiaticrus Fairmaire 1889: 41.
Athemus dimidiaticrus : Wittmer 1972a: 106. [Synonymized with *Athemus orientalis* (Gorham, 1889) by Wittmer 1995: 255. Secondary homonym, preoccupied by *Lycocerus dimidiaticrus* (Fairmaire, 1889: 40), originally in *Podabrus*.]
Lycocerus orientalis (Gorham, 1889): Kazantsev and Brancucci 2007: 252.

#### Type material examined.

*Telephorus dimidiaticrus*: Holotype ♂ (MNHN): [h]“Telephorus \ dimidiaticrus \ Fairm. \ Koui Tchéou”, [h]“Athemus \ prattianus \ (Gorh.) \ det. W. Wittmer”, [h]“Lycocerus \ dimidiaticrus (Fairm.) \ det. Y. X. Yang, 2009”, [p]“HOLOTYPUS”.

#### Additional material examined.

CHINA: Fujian: 1♂ (NHMB): “Fukien. Kuatun, 22.IV.1946”; 1♂ (NHMB): same locality, 1.V.1946; 1♀ (NHMB): same locality, 2.V.1946; 1♀ (IZAS): Jianyang, Huangkeng, Aotou, 950m, 5.V.1960, leg. Yong Zuo; 1♂ (IZAS): same locality, 800−1050m, 26.IV.1960, leg. Cheng-Lin Ma.

#### Distribution.

China (Fujian, Guizhou).

#### Etymology.

The new name is named after L. Fairmaire, the taxonomist who described this species.

#### Remarks.

Fairmaire described a *Podabrus dimidiaticrus* Fairmaire, 1889: 40 which became *Athemellus dimidiaticrus* by Wittmer (1972b) and now *Lycocerus dimidiaticrus* (Fairmaire, 1889), which however was neglected by Kazantsev and Brancucci (2007), and in the same original publication also a *Telephorus dimidiaticrus* Fairmaire, 1889: 41 which became *Athemus dimidiaticrus* by Wittmer (1972a) and now to be placed in *Lycocerus*, the two species become secondary homonyms and the junior is invalid (ICZN, 4^th^, article 57.31.), so that a new name is needed, *Lycocerus fairmairei* nom. n., to replace the name of the latter species.

At the same time, having examined the holotype of *Telephorus dimidiaticrus* and lectotype of *Lycocerus orientalis* (Gorham, 1889) (1♂ (NHMB): [p]“Foochau \ April, 1886 \ Leech.”, [h]“A. orientalis”, [p]“LECTOTYPE”, [p]“CANTHARIDAE \ CANTH00001609”.), we found that the formeris distinctly different from the latter in the aedeagus (Figs 19−21; the latter’s, see Wittmer 1995: figs. 109, 110), so we suggest *Telephorus dimidiaticrus* Fairmaire, 1889: 41, with its new replacement name *Lycocerus fairmairei* nom. n., to be resurrected from synonymy with *Lycocerus orientalis*.

### Lycocerus confossicollis

(Fairmaire, 1891) comb. n.

http://species-id.net/wiki/Lycocerus_confossicollis

Figs 10–11

Telephorus confossicollis Fairmaire 1891: ccviii.
Cantharis confossicollis : Jacobson 1911: 679.
Athemus (s.str.) *multiimpressus*Wittmer 1997: 285, figs. 128, 129.
Lycocerus multiimpressus : Kazantsev and Brancucci 2007: 252. syn. n.

#### Type material examined.

*Telephorus confossicollis*: Lectotype ♂ (MNHN): [h]“Changyang”, [h]“Telephorus \ confossicollis \ Fairm. \ Tchangyang”, [h]“Athemellus \ confossicollis \ (Fairm.) \ det. W. Wittmer”, [p]“LECTOTYPE”.

*Athemus* (s.str.) *multiimpressus*: Holotype ♂ (NHMB): [p]“Chin-ling Mts. \ Shensi. E. B. \ Apr.-May,1904”, [h]“Athemus \ multiimpressus \ Wittm. \ det. W. Wittmer”, [p]“HOLOTYPUS”, [h]“REM \ 95 \ 1314”, [p]“CANTHARIDAE \ CANTH00001208”.

#### Additional material examined.

CHINA: Hubei: 1♂ (IZAS): Xingshan, Longmenhe, 6.V.1994, leg. Xing-Ke Yang; 1♀ (IZAS): same data, 9.V.1994; 1♀ (IZAS): same locality, 9.V.1994, leg. You-Wei Zhang; 1♂ (IZAS): same locality, 730m, 22.VI.1994, leg. Jian Yao.

#### Distribution.

China (Shaanxi, Hubei).

#### Remarks.

Although the type specimen of *Cantharis confossicollis* was attached with Wittmer’s manuscript label “*Athem ellus confossicollis* (Fairm.)”, it has never been published formally for this taxonomic change. In our opinion, this speciesis definitely a member of *Lycocerus* due to the following characters: pronotum subquadrate, all tarsal claws simple and the aedeagus with dorsal plates of parameres separated. At the same time, *Lycocerus multiimpressus* (Wittmer, 1997) is considered to be a junior synonym of *Lycocerus confossicollis* (Fairmaire, 1891), comb. n., since we could not find differences between both nominal species in their morphological characters, including appearance and aedeagus. Therefore, we suggest *Lycocerus multiimpressus* is a new subjective synonym of *Lycocerus confossicollis*.

**Figures 10–15.**
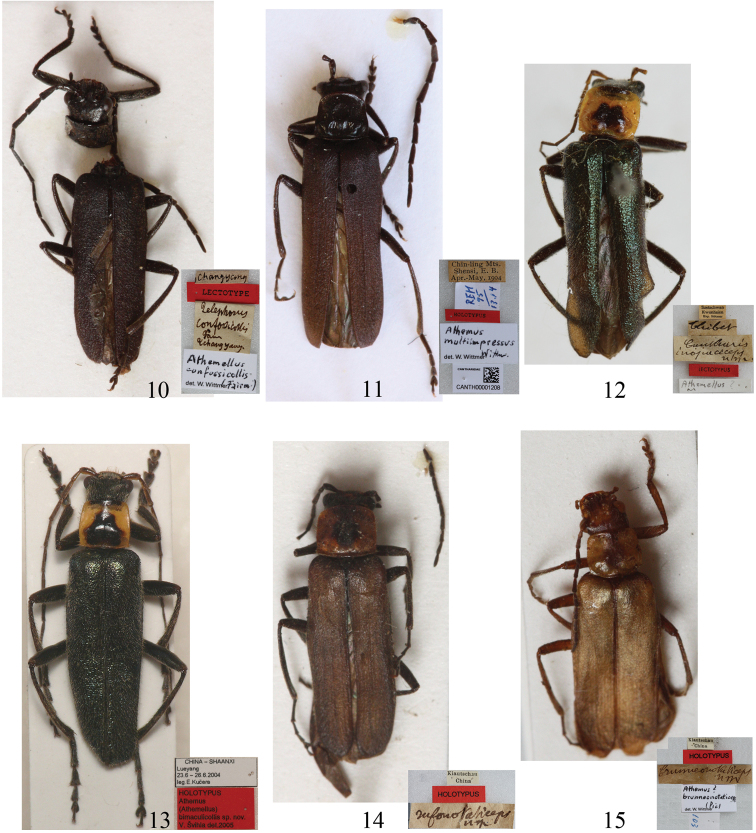
Habitus, dorsal view **10** Lectotypeof *Telephorus confossicollis* Fairmaire, 1891 **11** Holotypeof *Athemus* (s.str.) *multiimpressus* Wittmer, 1997 **12** Lectotypeof *Cantharis inopaciceps* Pic, 1926 **13** Holotypeof *Athemus (Athemellus) bimaculicollis* Švihla, 2005 **14** Holotypeof *Cantharis rufonotaticeps* Pic, 1921 **15** Holotypeof *Cantharis brunneonotaticeps* Pic, 1922. **10–11, 13** male **12, 14–15** female.

**Figures 16–23.**
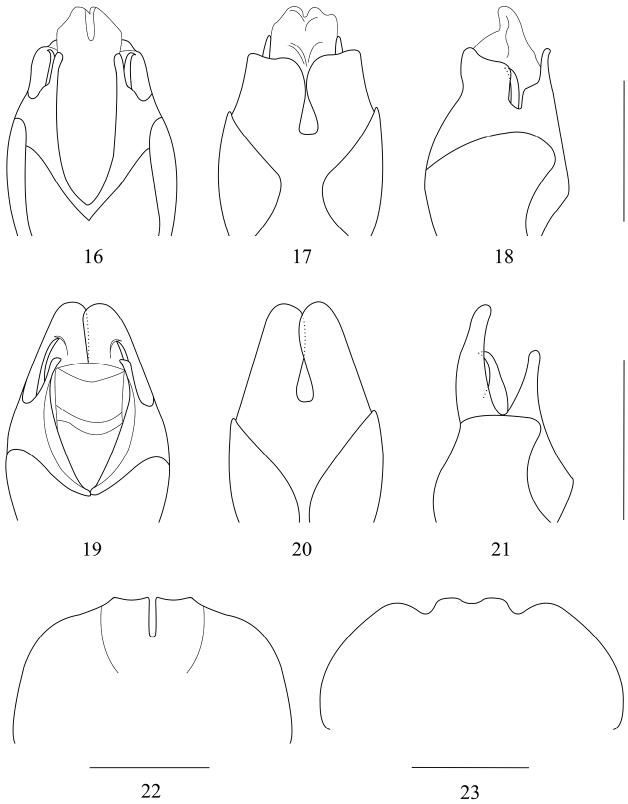
**16-21** Aedeagus (**16, 19** ventral view **17, 20** dorsal view **18, 21** lateral view) **22–23** female abdominal sternite VIII, ventral view **16–18** *Themus (Haplothemus) licenti* Pic, 1938 **19–21, 23** *Lycocerus dimidiaticrus* (Fairmaire, 1889) **22** *Themus (Haplothemus) coriaceipennis* (Fairmaire, 1889). Scale bars: 1 mm.

### Lycocerus inopaciceps

(Pic, 1926)

http://species-id.net/wiki/Lycocerus_inopaciceps

Figs 12–13

Cantharis inopaciceps Pic 1926b: 153.
Themus inopaciceps : Wittmer 1961: 362.
Athemellus inopaciceps : Wittmer 1983a: 190.
Athemus (Athemellus) bimaculicollis Švihla 2005: 90, figs 38–41. syn. n.
Lycocerus inopaciceps : Kazantsev and Brancucci 2007: 250.

#### Type material examined.

*Cantharis inopaciceps*: Lectotype ♀ (MNHN): [p]“Szetschwan \ Kwanhsien \ Exp. Stözner”, [h]“Thibet”, [h]“Cantharis \ inopaciceps \ n. sp.”, [h] “Athemellus ?”, [p]“LECTOTYPUS”.

*Athemus (Athemellus) bimaculicollis*: Holotype ♂ (NMPC): [p]“CHINA-SHAANXI \ Lueyang \ 23.6−26.6.2004 \ leg. E. Kučera”, [p]“HOLOTYPUS \ Athemus \ (Athemellus) \ bimaculicollis sp. nov.\ V. Švihla det. 2005”. Paratype: 1♀ (NMPC): same data.

#### Additional material examined.

CHINA: Shaanxi: 1♂, 1♀ (HBUM): Liuba, 10.−12.VI.2005, leg. Yi-Bin Ba; 1♂, 1♀ (HBUM): Liuba, Miaotaizi, 14.−15.VI.2005, leg. Yi-Bin Ba; 1♂, 4♀♀ (HBUM): Fengxian, Heigou, 13.VI.2005, leg. Yi-Bin Ba; 3♀♀ (HBUM): Liuba, Zaomulan, 11.VI.2005, leg. Yi-Bin Ba.

#### Distribution.

China (Shaanxi, Sichuan).

#### Remarks.

*Athemus (Athemellus) bimaculicollis* Švihla, 2005, which was omitted in the catalogue by Kazantsev and Brancucci (2007), is considered to be a junior synonym of *Lycocerus inopaciceps* (Pic, 1926), because we could not find any difference between their holotypes in the morphology including body size and coloration, tarsal claws and abdominal sternite VIII of female.

### Lycocerus nigratus

Y. Yang & X. Yang nom. n.

Podabrinus nigricolor Wittmer 1951: 99.
Pseudoabsidia nigricolor : Wittmer 1969: 128.
Athemellus nigricolor : Wittmer 1972b: 124.
Lycocerus nigricolor : Kazantsev and Brancucci 2007: 252. [Secondary homonym, preoccupied by *Lycocerus nigricolor* (Pic, 1938).]

#### Distribution.

China (Fujian).

#### Etymology.

The new specific name is derived from Latin word “*niger*” = black, a reference to its black body coloration, as the same meaning as its original name.

#### Remarks.

This species was originally described in *Podabrinus*, its original name is a junior secondary homonym of *Lycocerus nigricolor* (Pic, 1938) (originally in *Athemus*, located in Malaya), so it was replaced by *Lycocerus nigeratus* nom. n. (ICZN, 4^th^, article 57.3.1).

### Lycocerus plebejus

(Kiesenwetter, 1874)

http://species-id.net/wiki/Lycocerus_plebejus

Figs 14−15

Cantharis plebeja Kiesenwetter 1874: 278.
Lycocerus plebejus : Okushima 2005: 114–116, figs 11e, 12g, 14e, 32, 90.
Cantharis rufonotaticeps Pic 1921: 29. syn. n.
Cantharis brunneonotaticeps Pic 1922: 32.
Athemus brunneonotaticeps : Wittmer 1995: 268, fig. 199.
Lycocerus brunneonotaticeps : Kazantsev and Brancucci 2007: 249. syn. n.

#### Type material examined.

*Cantharis rufonotaticeps*: Holotype ♀ (MNHN): [p]“Kiautschau \ China”, [h]“rufonotaticeps \ n. sp.”, [p]“HOLOTYPUS”.

*Cantharis brunneonotaticeps*: Holotype ♀ (MNHN): [p]“Kiautschau \ China”, [h]“brunneonotaticeps \ n. sp.”, [h]“Athemus? \ brunneonotaticeps \ (Pic) \ det. W. Wittmer”, [p]“HOLOTYPUS”.

#### Additional material examined.

CHINA: Shanghai: 1♂ (NHMB): “China, Kiangsu Prov., Shanghai, 15.IV.1932, A. Bavio coll.”; 1♂, 1♀ (IZAS): “China, Kiangsu Prov., Shanghai, Zi-ka-wei, 20.IV.1924”. Jiangxi: 1♀ (MNHN): “Süd-China, Pingshiang, Dr. Kreyenberg”. Fujian: 1♂ (NHMB): “Fukien, Chungan, Bohea Hill, 15.III.1940, coll. T. C. Maa”; 1♀ (NHMB): same data, 16.III.1940; 1♂, 1♀ (NHMB): same data, 29.III.1940; 1♂ (NHMB): “Fukien, Shaowu, Shuipeikai, 3.IV.1942, coll. T. C. Maa”; 1♂ (NHMB): “Fukien, Shaowu, Kuhsienkai, IV.1944, coll. T. C. Maa”; 1♀ (NHMB): “Fukien, Chungan, Kuatun, IV.1942, coll. T. C. Maa”; 1♂ (IZAS): Shaowu, Chengguan, 150−220m, 19.IV.1960, leg. Cheng-Lin Ma; 1♀ (IZAS): same locality, 160−210m, 17.III.1960, leg. Yong Zuo; 1♀ (IZAS): same locality, 150−190m, 18.III.1960, leg. Yong Zuo. Guangxi: 1♂ (IZAS): Guilin, Liangfeng, 17.III.1952; 1♀ (IZAS): same locality, 26.III.1952. Sichuan: 1♀ (IZAS): Mt. Emei, Baoguosi, 550−750m, 7.IV.1957, leg. Ke-Ren Huang; 1♂ (IZAS): same locality, 12.IV.1957, leg. You-Cai Yu; 1♀ (IZAS): same data, 19.IV.1957; 1♀ (IZAS): same data, 3.IV.1957.

#### Distribution.

China (Shandong, Shanghai, Jiangxi, Fujian, Guangxi, Sichuan).

#### Remarks.

Having examined the holotypes of *Cantharis rufonotaticeps* Pic, 1921 and *Lycocerus brunneonotaticeps* (Pic, 1922) (originally in *Cantharis*), as well as a large series of additional specimens including both sexes, we were unable to find differences justifying their separation, although some variation in the coloration of head and elytra, which has led us to consider all the examined specimens of both nominal species to be conspecific. Furthermore, we discovered that their characters are consistent with the redescription and illustrations of *Lycocerus plebejus* (Kiesenwetter, 1874) provided by Okushima (2005). Consequently, we synonymized *Cantharis rufonotaticeps* and *Lycocerus brunneonotaticeps* under *Lycocerus plebejus*, whichconfirmed Okushima’s presum that the latter occurs in China but Japan.

### Lycocerus swampingatus

(Pic, 1916) comb. n.

http://species-id.net/wiki/Lycocerus_swampingatus

Figs 19−21

Cantharis swampingata Pic 1916: 4.

#### Type material examined.

*Cantharis swampingata*: Holotype ♂ (MNHN): [p]“Swamping \ China”, [h]“swampingata \ Pic”, [h]“n. sp.”, [h]“Lycocerus \ swampingatus(Pic) \ det. Y. X. Yang, 2009”, [p]“HOLOTYPUS”.

#### Distribution.

China (Sichuan).

#### Remarks.

The type specimen of this species was damaged seriously, lacking the abdomen, thorax, all legs, one elytron and part of head, but its aedeagus has been kept well, of which dorsal plates of parameres are separated (Figs 19−21), which is a diagnostic character of *Lycocerus* (Okushima, 2005). This species is similar to *Lycocerus canthariformis* (Ishida, 1986) (located in Japan) in the pronotum, which is rounded, wider than long, lateral margins are arcuate and posterior angles rounded, but the aedeagus is differs from that of the latter. Also, it is related to *Lycocerus pubicollis* (Heyden, 1889) in the aedeagus, but obviously different from the latter in the pronotum. Consequently, we suggest the following new combination: *Lycocerus swampingatus* (Pic, 1916), comb. n.

## Supplementary Material

XML Treatment for Themus (s.str.) regalis

XML Treatment for Themus (s.str.) scutulatus

XML Treatment for Themus (Telephorops) coelestis

XML Treatment for Themus (Telephorops) uniformis

XML Treatment for Themus (Haplothemus) licenti

XML Treatment for Lycocerus aenescens

XML Treatment for Lycocerus asperipennis

XML Treatment for Lycocerus borneoensis

XML Treatment for Lycocerus bilineatus

XML Treatment for Lycocerus fairmairei

XML Treatment for Lycocerus confossicollis

XML Treatment for Lycocerus inopaciceps

XML Treatment for Lycocerus nigratus

XML Treatment for Lycocerus plebejus

XML Treatment for Lycocerus swampingatus
